# Losing its ground: A case study of fast declining populations of a ‘least-concern’ species, the bonnet macaque (*Macaca radiata*)

**DOI:** 10.1371/journal.pone.0182140

**Published:** 2017-08-23

**Authors:** Joseph J. Erinjery, Shanthala Kumar, Honnavalli N. Kumara, K. Mohan, Tejeshwar Dhananjaya, P. Sundararaj, Rafi Kent, Mewa Singh

**Affiliations:** 1 Biopsychology Laboratory and Institute of Excellence, University of Mysore, Mysuru, India; 2 Department of Geography and Environment, Bar-Ilan University, Ramat Gan, Israel; 3 Department of Zoology, Bharathiar University, Coimbatore, India; 4 Sálim Ali Centre for Ornithology and Natural History, Coimbatore, India; 5 Jawaharlal Nehru Centre for Advanced Scientific Research, Bangalore, India; Centre for Cellular and Molecular Biology, INDIA

## Abstract

The populations of many species that are widespread and commensal with humans have been drastically declining during the past few decades, but little attention has been paid to their conservation. Here, we report the status of the bonnet macaque, a species that is considered ‘least-concern’ for conservation. We show that the widely ranging rhesus macaque is expanding its range into the distributional range of the bonnet macaque, a species endemic only to southern India. Bonnet macaques have very low abundance in forests of all types indicating that it is not a typically forest dwelling species. The traditionally preferred habitats of bonnet macaques have been Hindu temples/ tourist spots but our data reveal that nearly 50% population of bonnet macaques has disappeared from such previously occupied spots. Another preferred habitat of bonnet macaques has been roadsides with abundant *Ficus* trees adjoining croplands. We found that between 2003 and 2015, the roadsides have drastically changed where vegetation has been replaced with barren lands and urbanization. Consequently, the populations of bonnet macaques have declined by more than 65% over the past 25 years, and by more than 50% between 2003 and 2015 alone. We, therefore, conclude that this ‘least-concern’ species is actually facing serious conservation challenges. We also identify a few places such as small hillocks with natural vegetation and a few temples/tourist spots which are likely to remain stable and thus can serve as ‘bonnet macaque conservation reserves’. Since the bonnet macaque shares many traits with several other commensal and ‘low-risk’ species, it can serve as a model for the development of long-term conservation strategies for most such species.

## 1. Introduction

Many species of animals that are geographically widespread, largely commensal to humans, and found in relatively large numbers, are labelled as “least concern” in terms of conservation and their populations remain data deficient [1]. However, many such common species have undergone a drastic decline in their populations in the past few decades (e.g. House sparrow (*Passer domesticus*) see [2]; Starling (*Sturnus vulgaris*) see [3]; Long-tailed macaque (*Macaca fascicularis*) see[1]). Systematic population and ecological monitoring is required for a proper management of such species.

Several factors contribute to the decline of the commensal species. Most of these species compete with humans for resources, often resulting in human-animal conflict, which in turn, lead to killing of animals by people. Long-tailed macaques at temples in Bali are killed when they raid the nearby crops, despite being a protected species [4]. In India, primates are often harassed, trapped and relocated elsewhere even from the places of Hindu worship (M Singh–personal observations).A drastic decline of a commensal species may also occur due to habitat fragmentation following land use modification, urbanization [5], increased predation pressure [6], elevated parasite loads [4] and interspecific competition [5]. Land use modifications caused by human activities have led to the extinction of several animal species [7–13]. Land use modifications lead to habitat loss by destruction of the vegetated areas, habitat degradation by reduction in quality of vegetated areas, habitat isolation by reduced land use connectivity, and changes in biology, behaviour and interactions of a species [9]. One of the main drivers of land-use modification is infrastructure development [14,15], a process of urbanisation, which is a consequence of expanding human population [8]. Populations of several species have been shown to utilize remaining patches of intact vegetation on roadsides or places of human worship in the human modified landscapes as marginal habitats (e.g. Lion-tailed macaques, *Macaca silenus* [16]; Willow warblers, *Phylloscopus trochilus* [17]; Bank vole, *Clethrionomys glareolus* [18]). Interspecific interaction with predators and competitors resulting in reduction of distributional range has also been shown to be a major reason for the decline and extinction of several species [19,20].Mammals are found to be extremely vulnerable to these conditions [8,14]and their responses to changing land use can serve as a good indicator of adaptability or local extinction for other vertebrates and invertebrates inhabiting the same area.

Since habitats of most commensal and apparently ‘abundant’ species are undergoing rapid changes everywhere, it is necessary to pay attention to their conservation and management before such species become threatened. Here we present the bonnet macaque (*Macaca radiata*) as a representative example of such a species and show why this so-called ‘least-concern’ species actually requires urgent conservation attention.

Since the challenges faced by the commensal species are manifold, a multidimensional approach is required to properly assess the populations and conservation status of such species, as is presented here. The bonnet macaque is a largely commensal, habitat generalist species of macaques, endemic to south India [21]. It is listed as ‘least concern’ by IUCN [22]. The preferred habitat of the bonnet macaque is human dominated landscapes [23,24], especially along vegetated roadsides [25,26]. It is not known since how long this change in habitat preference has been there but the forest and the non-forest bonnet macaques differ in several traits. The forest monkeys have a smaller group size and spend less time on movement and social behaviour than the rural and urban monkeys [27]. Individuals often come into conflict with humans in rural and urban areas [26]. The conflicts with humans have led to injuries, and unplanned translocations and killings of macaques [25]. Previous studies on bonnet macaques have shown that there is a decline in their populations in several habitats [25,26] and that populations of bonnet macaques are in low density in forest habitats [26].Another recent study has shown that the distributional range of bonnet macaques is declining due to a range extension by rhesus macaques (*Macaca mulatta*)[20] in peninsular India. As the nature of habitats and animal-human interactions in many animal species are apparently similar to those described above for the bonnet macaque, we propose that the bonnet macaque can serve as a model species for conservation of other commensal species.In order to assess the current status of this species, we set out to conduct a study with a multifold approach, and made certain specific predictions including (i) the widespread rhesus macaques are extending their range and pushing the distributional range of bonnet macaques southwards in peninsular India, (ii) the bonnet macaques are found in low densities in the forest areas that are reserved for wildlife conservation, (iii) the number of bonnet macaques is declining in temples/tourist spots where they receive food from humans, and (iv) the number of bonnet macaques is drastically declining from roadside habitats where they obtain required food primarily by raiding crops in the surrounding agricultural fields. Finally, we discuss conservation management strategies for the ‘least concern’ commensal bonnet macaques.

## 2. Methods

### 2.1 Ethics statement

We followed all national and international ethical guidelines during this research. Proper permits were obtained from concerned Forest Departments of the states of Karnataka, Tamil Nadu and Kerala for research in Protected Areas. We obtained some information through interviews with local residents, temples and forest authorities regarding presence/absence of monkeys, and since this interaction did not involve any ‘sample collection’, ‘intervention’ or ‘invasion of privacy’, a clearance from Institutional Human Ethics Committee was not required as per regulations. Since no personal data or identities of these informants were asked and their replies were only noted down and not tape recorded, we felt that an oral consent was sufficient and hence, we did not obtain any written consent.

### 2.2 Range extension of rhesus macaques into the distribution range of bonnet macaques

Kumar et al. [20]conducted a survey between 2004 and 2008 on populations of rhesus and bonnet macaques across interspecific border zones in peninsular India and reported that rhesus macaques have extended their range by about 3500 km^2^ into the range of bonnet macaques when compared to earlier range drawn by Fooden et al. [28] and Koyama and Shekar[29] in 1981. We conducted further surveys in the same region in 2014 and 2015 to assess whether there was any further range extension by rhesus macaques. The surveys were conducted on the roadsides adjoining the previous southern most boundary of rhesus macaque described by Kumar et al. [20]. The total distance surveyed was 1140 km. We also confirmed with local residents and the Forest Department personnel whether the groups were naturally occurring or were translocated. These individuals were orally informed that the information we required was only about the presence/absence of monkey groups, and if any monkey group was translocated, the place and date of capture of the group. No personal data or even their identities were asked. The replies were noted down and not tape recorded. These informants were told that the information so obtained will be used only for the purpose of this research study and will not be revealed elsewhere. Since the information asked from the people did not involve ‘personal data’, ‘individual identities’, ‘intervention’, ‘sample collection’ and ‘invasion of privacy’, we did not apply for clearance from the Institutional Human Ethics Committee. We drew the southern boundary of rhesus macaque by connecting different GPS locations of the groups found in the southernmost part of a particular longitude spanning across the width of the Indian peninsula and calculated the area of extension using GIS. We did not include introduced groups while drawing the boundary since such groups are demographically unstable and remain nomadic for long periods of time.

### 2.3 Occupancy modelling of bonnet macaques in forest areas

**Fig 1:**
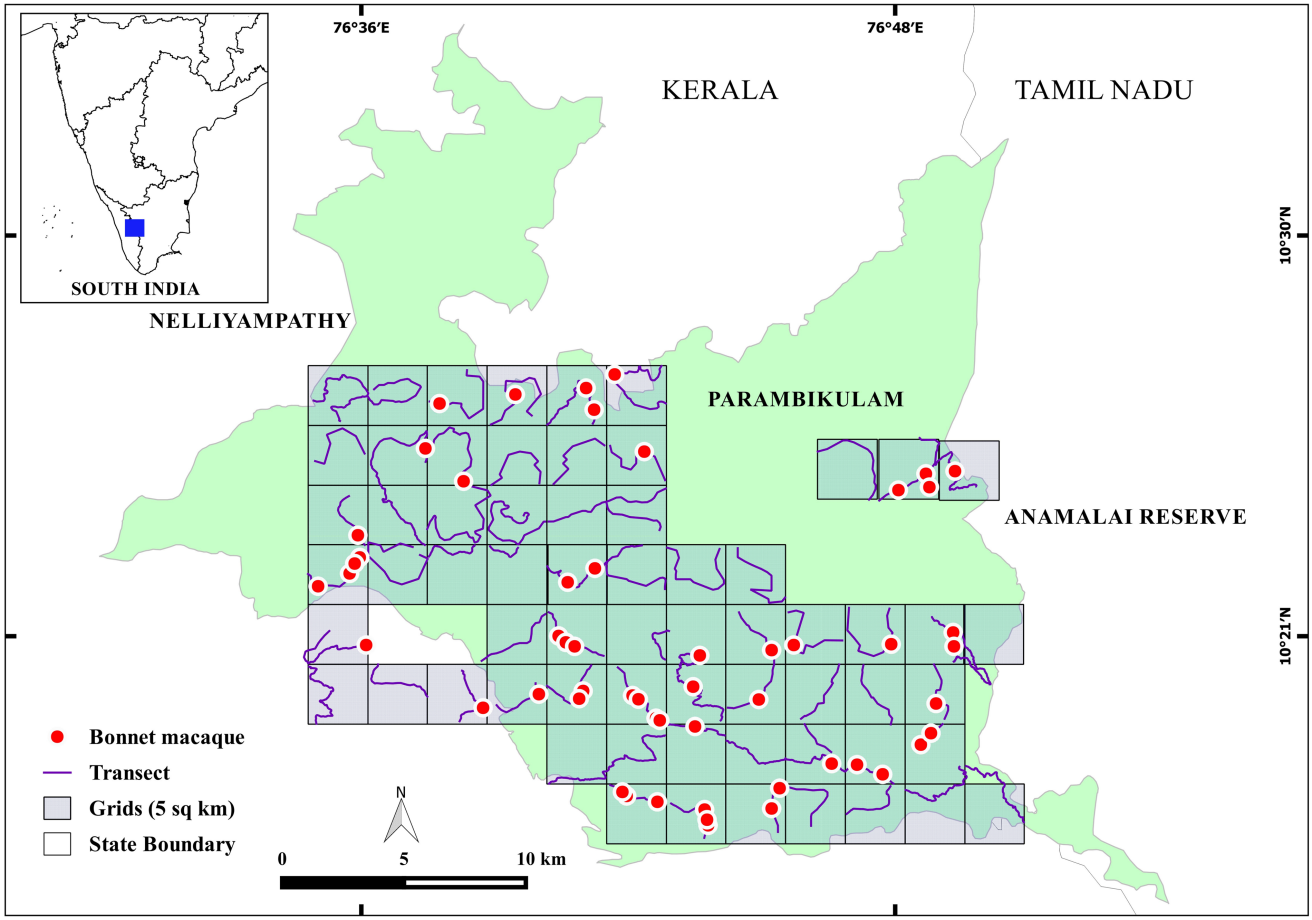
Locations of line transects and bonnet macaque groups in the Parambikulam landscape.

We conducted a study in the Parambikulam Tiger Reserve, (10°20′–10°26′N and 76°35′–76°50′E) in the state of Kerala between October 2010 and May 2012. The major vegetation types in the area include the west coast tropical evergreen forest, the west coast semi evergreen forest, the mixed moist deciduous forest and the mixed dry deciduous forest [30,31]. We laid 5 km^2^ grids over the Parambikulam landscape and based on the vegetation cover, we selected 64 grid cells for the present study (Fig 1). In each grid cell, we made four replicated walks using existing trails or animal paths of lengths varying between 2–7 km, depending on the terrain and feasibility. The total distance walked over the course of the study was 1098 km. Since the study area is a protected tiger reserve with little human movement, we presumed that the habituation level of the macaque groups would be the same in all grid cells.Whenever a group was encountered around the trail, we recorded group size, group location and age/sex composition. By pooling the data from the replicated walks, we calculated the mean number of groups encountered per km of each trail.

We collected data on height of the tallest trees and human disturbance index at regular intervals of 250 m as site covariates. The heights of the ten tallest trees were measured using Nikon Forestpro Laser Rangefinder and we consequently computed mean height of the tallest trees for all locations in a grid. We categorized human disturbance as low (total score: ≤6) intermediate (total score: 7–15) and high (total score: ≥16), based on data collected on human movement, lopping, grazing and presence of stumps in each grid cell. A ten-point scale was used to measure human movement and lopping at each location and the scoring was based on the percentage of locations with these signs (0:0%; 1:10%; 2: 20%………………..10: 100% of locations). We recorded grazing and presence of stumps as Yes or No: Yes = 4; No = 0).Further, we recorded forest type, altitude and the Normalized Difference Vegetation Index (NDVI) in each grid cell using GIS and available maps of the area, which were also used as site covariates.

### 2.4 Occupancy modelling

Detection histories for all the grid cells from four replications were analyzed using single season models in PRESENCE -ver. 3.0 [32]. The data from each replication were considered as one sample. We recorded the detection of bonnet macaques as 1 and non-detection as 0. We used likelihood functions [33] to estimate detection probability (ρ) and probability of occurrence of the bonnet macaques inside a grid cell (*ψ*). The length of the trail and the duration of walking were considered as covariates for detection probability. We considered mean height of the tallest trees, human disturbance index, percentage of evergreen forest, altitude and NDVI in a grid cell as site covariates for occupancy of the species in the grid cell. A logistic model with logit link and binomial error was performed to estimate the effect of covariates on detection probability (ρ) and on occupancy (*ψ*), following Burnham et al. [34]. Additionally, we compiled the information on the population status of bonnet macaques from the available literature from the states of Karnataka and Tamil Nadu from different forest types.

### 2.5 Survey of temple sites/tourist spots

Kumara et al. [26] had previously conducted a study on the temple/tourist spot populations of bonnet macaques in 26 districts of the state of Karnataka (11°31′–18°45′N and 74°12′–78°40′E) from 2001 to 2004. The present study was conducted between November 2009 and September 2015 using the methodology employed by Kumara et al. [26]. The total distance covered on roads assessing these populations was 9697 km and we calculated the encounter rates per 100 km. We used encounter rates to compare populations between the present and past studies. We also conducted informal interviews with local residents and temple authorities to gather information about presence/absence of monkeys in the past (see Section 2.2 above).

### 2.6 Roadside survey

To observe the impact of human dominated landscapes on bonnet macaque populations, our research group has continuously, for over two decades, monitored populations of bonnet macaques inhabiting major roadsides which connect the city of Mysore in southern India to other major towns. We defined a roadside as an area outside the road which is within 15 m from the centre of the road on both sides, based on observations made during the present and previous studies [25,35–37]. Previous surveys, conducted in 1989, 1998, 2003 and 2009 on the populations of bonnet macaque concluded that the annual decline in the population of bonnet macaques was more pronounced between 2003 and 2009 (4.0%) when compared to annual population decline rate obtained between 1989 and 2003 (1.5%). Loss of roadside trees and increase in urbanisation due to the widening of roads after 2004 was speculated as the major reason for the decline of the populations in these surveys [25,26]. Considering these aspects, we re-surveyed these major roadsides in 2015 to evaluate whether these populations are still declining at a similar rate as observed in 2003–2009 and whether the bonnet macaque populations are affected by land use modifications between 2003 and 2015 along the previously surveyed roads.

The study included five highways connecting the city of Mysore to other major towns, one adjacent road connecting the highways and one scrub forest road at Chamundi Hill near the city. Roadsides were usually bordered by agricultural lands, urban area or scrub forest [25]. The roadside surveys took place between January and February 2015. The total distance surveyed was 464 km. Surveys were conducted by foot and on a motorcycle/car at speeds of <10 km h^-1^. We confirmed the location of each macaque group on the roadside using repeated surveys. Since the location of each macaque group was available from previous studies, we used the ‘total counts’ method. If a group was not found within 1 km of its location in the earlier surveys, we thoroughly searched the areas around the roadsides to confirm whether the groups actually disappeared or not. We further confirmed the disappearance of macaque groups by enquiring with farmers and villagers in the locality. We calculated density km^-1^ along each roadside by dividing the total number of animals counted along a roadside by the total distance covered on a roadside since the survey used linear transects.

### 2.7 Land use classification along the roadsides

We used high resolution remotely sensed Google Earth (Digital Globe; CNES/ Astrium) images for classification of different land use types along roadsides. To represent the status in 2003, we used images from the period between 2000 and 2006, since historical data from 2003 was not consistently available. We consistently used the earliest image possible for drawing the polygons. We omitted the Begur-Handpost road from these analyses, since images of this roadside were available only after 2010. We used images from 2015 to represent the 2015 period. We drew polygons representing different land use types on the clipped area of the roadside overlaid on the Google Earth image. If the roadsides were flattened for road construction, we considered that area as part of the road and not roadside. The different land use types considered for the classification were: vegetation (trees), urban area (man-made structures like buildings, roads, and aqueducts), barren area (area with no man-made structures) and water body. Geometric corrections were done after classification using ArcGIS 10.1. Further, we estimated the area covered by each land use type using ArcGIS 10.1.

### 2.8 Canopy connectivity

We drew polygons over connected canopies observed from the Google Earth images to measure the continuous area under canopy in 2003 and 2015. We considered an area under the canopy as continuous only if a canopy from one side of the road overlapped with canopy from the other side. The maps that were used for measuring canopy connectivity were the same as those used for the land use classification along the roadsides.

### 2.9 Land use classification around the roadsides

We classified land use around roadsides to determine whether the macaques have suitable habitats nearby, other than roadside habitat. We defined the area around roadside as area within 1 km on both sides from centre of the road. Since, the area around roadsides was large for doing manual classification, we used remotely sensed Landsat 7 scenes obtained from USGS (http://earthexplorer.usgs.gov/) to classify different land use types around the roadsides. Images acquired between January and April was used to obtain cloud free data. To represent the 2003 period, we obtained images from 2001 since no cloud free images were available in 2003. We could obtain Landsat images from 2015 to represent the 2015 period. Supervised classification was performed to classify different land use types using ERDAS IMAGINE 2013. The different land use types considered for classification were: vegetation, urban area, agricultural land and water body. All classified maps yielded an overall accuracy greater than 80%.We could not differentiate between agricultural land and barren area using the images.

## 3. Results

### 3.1 Range extension of rhesus macaques into the range of bonnet macaques

**Fig 2:**
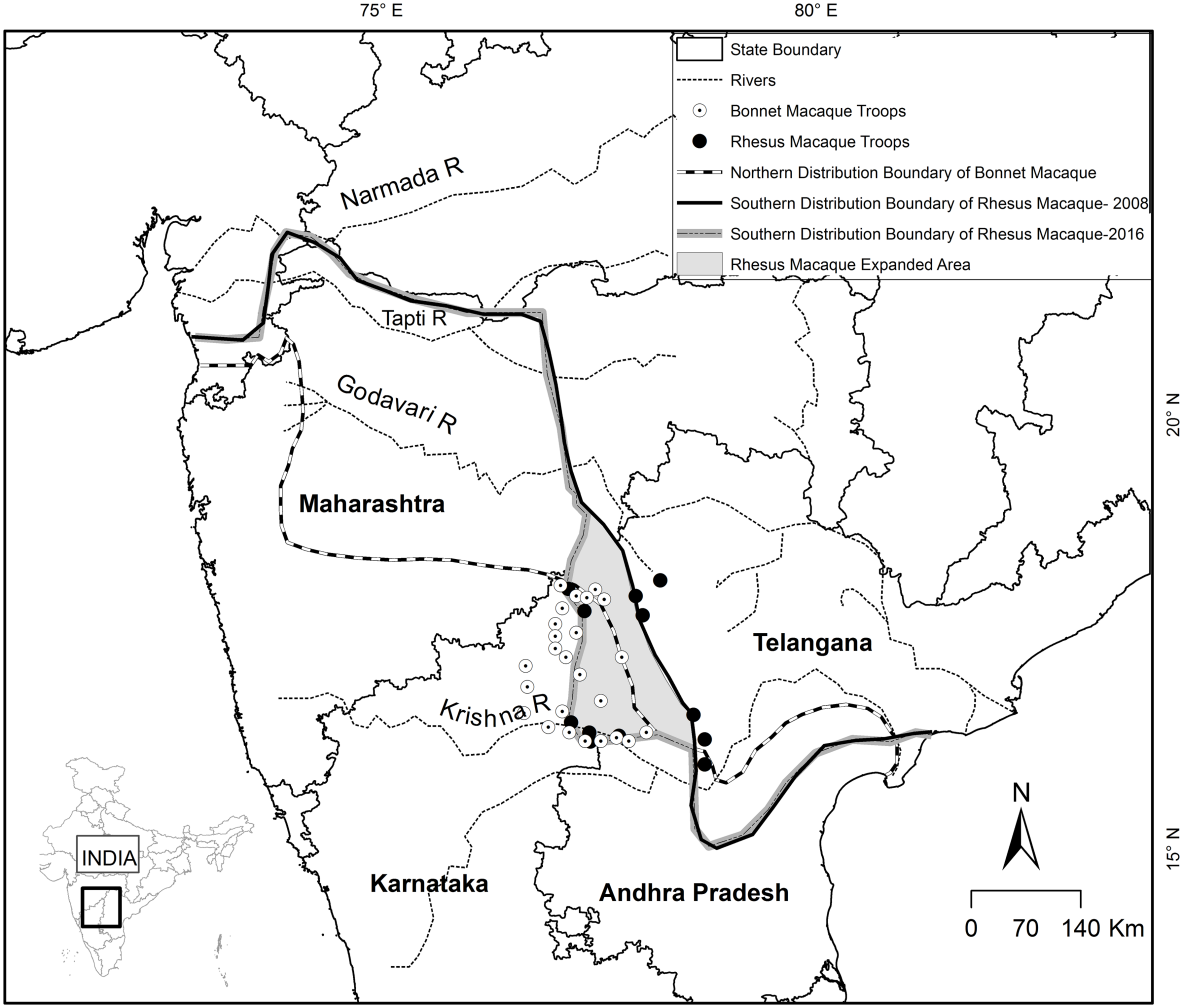
Range extension of the rhesus macaque at its southern distribution range limit. (Adapted and modified from Kumar et al. 2011[20]).

A total of thirty five groups of bonnet macaque and twelve groups of rhesus macaque were recorded (Table 1) in northern Karnataka and adjoining Telangana states. The encounter rate for bonnet macaques (0.03 groups/ km) was three times higher than that for rhesus macaques (0.01 groups /km). The mean group size of bonnet macaque was 12.3 ± 6.2 and of rhesus macaque was 9.8 ± 3.7 (Table 1, S1 Table). Among the twelve groups of rhesus macaques, six groups fell outside the earlier southern distributional range of the species (Fig 2). This amounted to a further range extension by rhesus macaques by 24,565 km^2^after that reported by [20]. The first prediction that the distributional range of bonnet macaques is further shrinking due to the range invasion by the rhesus macaque in peninsular India, therefore, is supported.

**Table 1:** Number of groups encountered and group parameters during the 1140 km of sampling in northern Karnataka and adjoining Telangana states.

Population parameter	*Macaca radiata*	*Macaca mulatta*
Number of groups	35	12
Number of groups/km	0.03	0.01
Mean group size (N)	12.3 ± 6.2 (6)	9.8 ± 3.7 (5)
Group size range	5–21	5–15

### 3.2 Occupancy modelling of bonnet macaques in forest areas

We recorded 54 groups of bonnet macaque in 33 grid cells of 64 grid cells sampled in the state of Kerala (S2 Table). The estimate of detection probability ($\hat{p}$) was 0.25±0.05_SE_, which indicates about 25% chance of detecting bonnet macaques if they were present in the area. The trail length or the duration of walk did not influence the detection probability (S3 Table). Subsequent models were run without any covariates as function of *p*.

The constant model (*ψ* (.), *p* (.) was the highest ranked model (ΔAIC_c_ = 0, *w*_*i*_ = 0.3973) among all the models (S4 and S5 Tables). None of the covariates influenced the occupancy of bonnet macaque. Thus occupancy estimates from all models were averaged and considered as the final estimate. Mean occupancy estimate was $\hat{\psi }$ = 0.51±0.08_SE_.

$\hat{p}$: is the estimated species detection probability; AICc: AIC corrected for small-sample bias; ΔAIC (.), (ELE), (EG), (ELE+EG), (ELE+CANO), (CANO), (DISTU), (EG+ CANO), (DISTU+ELE), (ELE+ CANO +EG), (DISTU+EG), (DISTU+ELE+ CANO), (DISTU+ CANO), (DISTU+ELE+ CANO+EG), Estimated occupancy parameter; S: Associated standard error; AICc: AIC corrected for small-sample bias; ΔAIC: difference in AICc values between each model and the model with the lowest AICc.

Although none of the covariates significantly influenced the occupancy estimate, the elevation of the grid (ΔAIC_c_weight = 0. 33) influenced the occurrence more than other covariates (Table 2). Occurrence of bonnet macaque was negatively correlated with elevation (*β* = -2.95±2.24_SE_), proportion of evergreen forest in the grid (*β* = 2.21±2.05_SE_) and canopy height (*β* = 1.70±2.09_SE_).

**Table 2:** Covariates influencing the bonnet macaque occupancy ranked on the basis of summed model weights of covariates, with beta coefficient and associated standard error of the models more than 2 ΔAIC_c_.

Covariate	Summed AIC_c_ weights	*β* coefficients	S$\hat{E}$
ELE	0.33	-2.95	2.24
EG	0.27	-2.21	2.05
CANO	0.24	-1.70	2.09
DISTU	0.17	0.18	1.93

EG: proportion of evergreen forests; ELE: elevation range; CANO: height of the canopy; DISTU: disturbance index

### 3.3 Bonnet macaques in different forest types

A summary of the data compiled from various studies in different forest types regarding the occurrence of bonnet macaques is shown in Table 3. The density estimate was available only for two protected areas i.e. 6.56 macaques/ km^2^ in Biligiri Rangaswamy Temple TR and 12.40 macaques/ km^2^ in Aghanashini LTM-CR. Despite intensive sampling in some of the protected areas, the scarcity of detections did not allow density estimation (e.g. Bhadra TR:[38], Kalakad-Mundanthurai TR:[39]. Only encounter rate was available for all other protected areas. Encounter rate varied from 0.011 to 0.208 macaques/ km. The mean encounter rate of bonnet macaque in protected areas was 0.077±0.063_SD_ that varied from 0.011 to 0.208 macaques/ km. However, in eleven of the sixteen protected areas, encounter rate was lower than the mean, and about 40% of the protected areas showed the encounter rate to be less than 0.05. The results, therefore, provide evidence for our second prediction that bonnet macaques are found in low densities in forest areas that are protected for wildlife conservation.

**Table 3:** Abundance of bonnet macaque groups encountered in different protected areas of Karnataka.

State	Name of PA/RF	Forest type	No. of km	No. of groups	Groups/km	Density	Source
Goa	Mollem NP, Bhagwan Mahaveer WLS, Bondla WLS, Mhadei WLS, Cotigao WLS, Netravali WLS	EGF+ MDF +DDF	149	11	0.073	-	Senguptha and Radhakrishna[40]
Karnataka	Biligiri Rangaswamy Temple TR	EGF+ MDF +DDF	795	43	0.030	6.56	Kumara et al.[41]
Karnataka	Bannerghatta NP	DDF+SCR+PLA	170	2	0.011	-	Kumara et al. [42]
Karnataka	Bandipur TR	MDF+DDF	245	8	0.033	-	Kumara et al. [26]
Karnataka	Nagarahole TR	MDF+DDF	261	9	0.034	-	Kumara et al. [26]
Karnataka	Brahmagiri WLS	EGF	118	8	0.068	-	Kumara et al. [26]
Karnataka	Talakaveri WLS	EGF	302	23	0.076	-	Kumara et al. [26]
Karnataka	Pushpagiri WLS	EGF	184	6	0.037	-	Kumara et al.[26]
Karnataka	Kudremukh NP	EGF	526	14	0.026	-	Netalkar and Kumara[43]
Karnataka	Someshwara WLS	EGF+AG	63	8	0.126	-	Netalkar and Kumara [43]
Karnataka	Mookambika WLS	EGF+AG	93	18	0.193	-	Netalkar and Kumara[43]
Karnataka	Bhadra TR	EGF+ MDF +DDF	473	Less detection	-	Could not estimate	Jathanna et al. [43]
Karnataka	Sharavathi Valley WLS	EGF+AG	186	31	0.167	-	Kumara et al.[26]
Karnataka	Aghanashini LTM-CR	EGF+AG	481	100	0.208	12.40	Bapureddy et al.[44]
Tamil Nadu	Indira Gandhi WLS	EGF+ MDF+DDF+SCR+PLA	185	12	0.066	-	Kumar et al. [45]
Tamil Nadu	Meghamalai WLS	EGF+ MDF +DDF+SCR+PLA	204	18	0.088	-	Kumara et al. [42]
Tamil Nadu	Kalkad-Mundnthurai TR	EGF+ MDF +DDF+SCR	353	<5	0.011	Could not estimate	Ramesh et al.[39]

NP: National Park; WLS: Wildlife Sanctuary; TR: Tiger Reserve; CR: Conservation Reserve

### 3.4 Bonnet macaques in temple sites/tourist spots

Table 4 presents the information on status of bonnet macaques at temples and tourist locations. Bonnet macaques have been translocated from temples and tourist locations in all three southern states of India. Currently, macaques are found in only about 31% of such locations. Whereas macaques have reappeared following translocations in about 20% of the cases, they have been eliminated or have disappeared from more than 48% of the locations. The results therefore provide evidence for our third prediction that the number of bonnet macaques is declining in temples/tourist spots which now do not appear to be stable habitats for these macaques.

**Table 4:** Population status of bonnet macaque at temples or tourist locations.

State	No. of sampling	Present or no report of relocation (%)	Translocated but reappeared (%)	Totally eliminated or no recent sightings (%)	Source
Karnataka	107	37 (34.58)	19 (17.76)	51 (47.66)	Kumara et al. [26]
Tamil Nadu	11	2 (18.18)	4 (36.36)	5 (45.45)	Current study
Kerala	6	0	2 (33.33)	4 (66.66)	Current study
Total	124	39 (31.45)	25 (20.16)	60 (48.38)	

### 3.5 Bonnet macaques on roadside habitats

#### 3.5.1 Population dynamics

Only 46% of the bonnet macaque population observed in 2003 remains on roadsides as per the survey of 2015 (S6 Table).The number of groups has significantly declined from 42 in 2003 to 24 in 2015 (chi-square test: χ^2^ = 8.76, P = 0.003). Similarly, the number of individuals declined from 889 in 2003 to 407 in 2015 (chi-square test: χ^2^ = 357.01, P < 0.001). Density in each roadside population was higher in 2003 than in 2015 (Wilcoxons signed rank test: Z = -2.37, P = 0.02). Overall population decline rate was 4.52 macaques year^-1^ (Table 5). The lowest decline rate was observed in Mysore–Kollegal roadside population (0.47 macaques year^-1^) and the highest decline rate was observed in Mysore-Ramnagar roadside population with total population extinction. The predicted total number of macaques after ten years in these roadsides is 256. Many roadside populations are expected to approach “near extinction” in next ten years (Table 5). Mean group size did not show any significant difference between 2003 (21.1 ± 12.2_SD_) and 2015 (17.1 ± 12.9 _SD_), due to large variations in the group size in both surveys (Mann- Whitney U test: U = 389.5, P = 0.13). However, the proportion of groups of small size (1–10 individuals) was higher in 2015 (43%) than in 2003 (24%; Chi-square test: χ^2^ = 7.27, P = 0.007). Overall, nearly 65% of the population of bonnet macaques on these roads has disappeared during the past 25 years (Table 6). Since the population on Chamundi Hill Road has remained stable, the total decline on all the other roads has been 78%.

**Table 5:** Population dynamics of bonnet macaque populations along different roadsides.

Name of the road	Distance covered (km)	Density/km		No. of animals/ No. of groups		Annual decline rate (%)	Expected population after 10 years*	Total population decline (%)
		2003	2015	2003^#^	2015			
Mysore-Ramnagar	91	1.85	0	168/8	0/0	-	0	100
Mysore-Kanakapura	98	0.47	0.31	46/3	30/3	2.89	15	35
Mysore-Kollegal	67	1.87	1.76	125/6	118/8	0.47	113	6
Mysore-Hangala	75	2.27	0.43	170/10	32/4	6.76	16	81
Mysore-HD Kote	70	0.6	0.1	43/4	7/1	6.97	3	84
Chamundi Hill	19	14	11	266/7	209/7	1.79	175	21
Begur-Handpost	44	1.61	0.25	71/4	11/1	7.04	5	84
TOTAL	464	1.92	0.88	889/42	407/24	4.52	256	54

^#^- Singh and Rao, 2004 [37]

*- Calculated using exponential decay rate formula.

**Table 6:** Status of bonnet macaque along road network of Mysore.

Sector	Distance covered (km)	No. of groups					No. of individuals				
		1989^1^	1998^2^	2003^3^	2009^4^	2015	1989	1998	2003	2009	2015
Mysore-Antharsanthe	70	7	7	4	2	1	113	116	43	26	13
Handpost_Begur	44	3	3	4	2	1	62	48	71	27	11
Mysore-Hangala	75	11	10	10	5	4	192	157	170	132	35
Nanjangud-Chamarajanagar	39	2	3	1	0	0	40	50	21	0	0
Chamarajanagar-Yelandur	43	1	2	2	0	0	28	60	11	0	0
Kollegala-T.Narsipuara	67	9	8	6	7	8	257	169	125	102	118
Mysore-Ramanagaram	91	7	8	8	1	0	133	103	168	34	0
Ramanagaram-Kanakapura	28	0	1	1	2	1	0	12	16	19	14
Mysore-Kanakapura	98	6	5	3	4	3	166	95	46	111	30
Mysore-Periyapatna-Nagarahole	77	1	1	1	0	0	6	6	13	0	0
Chamundi Hills Road	19	7	7	7	8	7	210	206	266	246	210
Total	651	54	55	47	31	25	1207	1022	950	697	431

^1^D’souza and Singh [35];

^2^Sharma [36];

^3^Singh and Rao [37];

^4^Singh et al. [25]

#### 3.5.2 Land use change along the roadsides

Land use along roadsides in 2003 was dominated by barren area (47%), followed by vegetation (39%), urban area (13%) and water bodies (1%). Land use along the roadsides in 2015 was dominated by urban area (55%), followed by barren area (27%), vegetation (18%), and water body (0%). There was a decrease in percent area covered by barren area (Wilcoxons signed rank test: Z = 2.21, P = 0.03) and vegetation (Wilcoxons signed rank test: Z = 2.20, P = 0.03) along all surveyed roadsides between 2003 and 2015 (Table 7). However, there was an increase in urban area along all the roadsides between 2003 and 2015 (Wilcoxons signed rank test: Z = 2.20, P = 0.03).

**Table 7:** Area covered (%) by each land use type along different roadside habitats.

Name of the road	Barren area (%)			Urban area (%)			Vegetation (%)			Water body (%)		
	2001	2015	Change*	2001	2015	Change	2001	2015	Change	2001	2015	Change
Mysore-Ramnagar	35	32	-29	21	52	+60	44	16	64	0	0	0
Mysore-Kanakapura	53	45	-15	13	30	+56	34	25	-26	0	0	0
Mysore-Kollegal	52	43	-17	10	22	+54	37	34	-8	1	1	0
Mysore-Hangala	49	13	-73	11	81	+86	39	6	-85	1	0	-100
Mysore-HD Kote	58	14	-15	13	77	+83	29	9	-69	0	0	0
Chamundi Hill	12	4	-58	5	14	+64	83	82	+1	0	0	0
Total	47	27	-48	13	55	+76	39	18	-54	1	0	-100

*(+)—Increase, (-)–decrease

**Fig 3:**
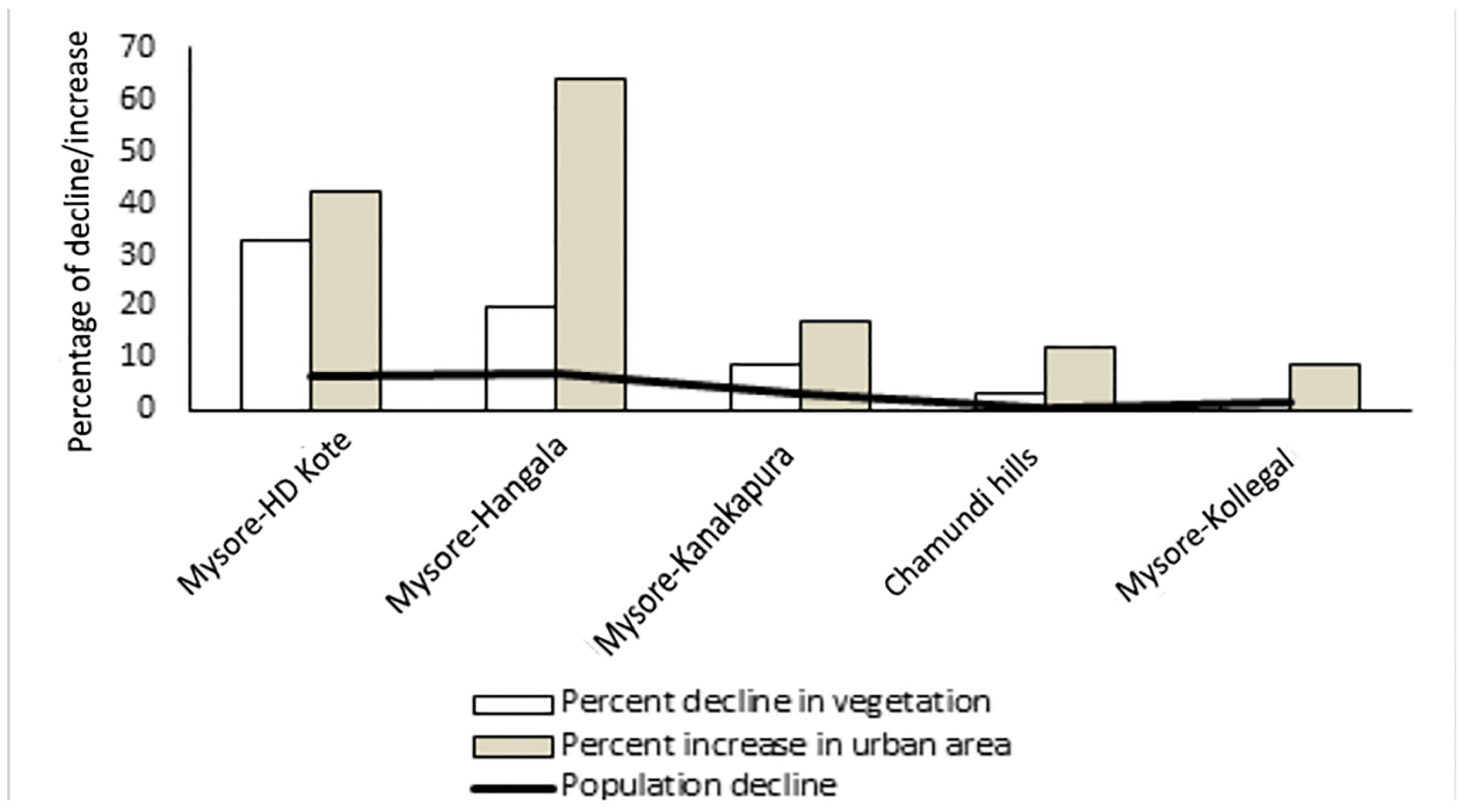
Vegetation, urban area and percentage of annual population decline of macaques on the roadsides.

Decline in vegetation (%) and increase in urban area (%) were good predictors of annual population decline (%) along the different roadsides between 2003 and 2015 (linear regression: R^2^ = 0.919, β = -0.075, P = 0.01; R^2^ = 0.873, β = 0.183, P = 0.02; Fig 3). Vegetation (%) along roadsides predicted the density of animals both in 2015 (linear regression: R^2^ = 0.791, β = 0.022, P = 0.01) and 2003 (linear regression: R^2^ = 0.877, β = 3.08, P = 0.006).

#### 3.5.3 Canopy connectivity loss

**Fig 4:**
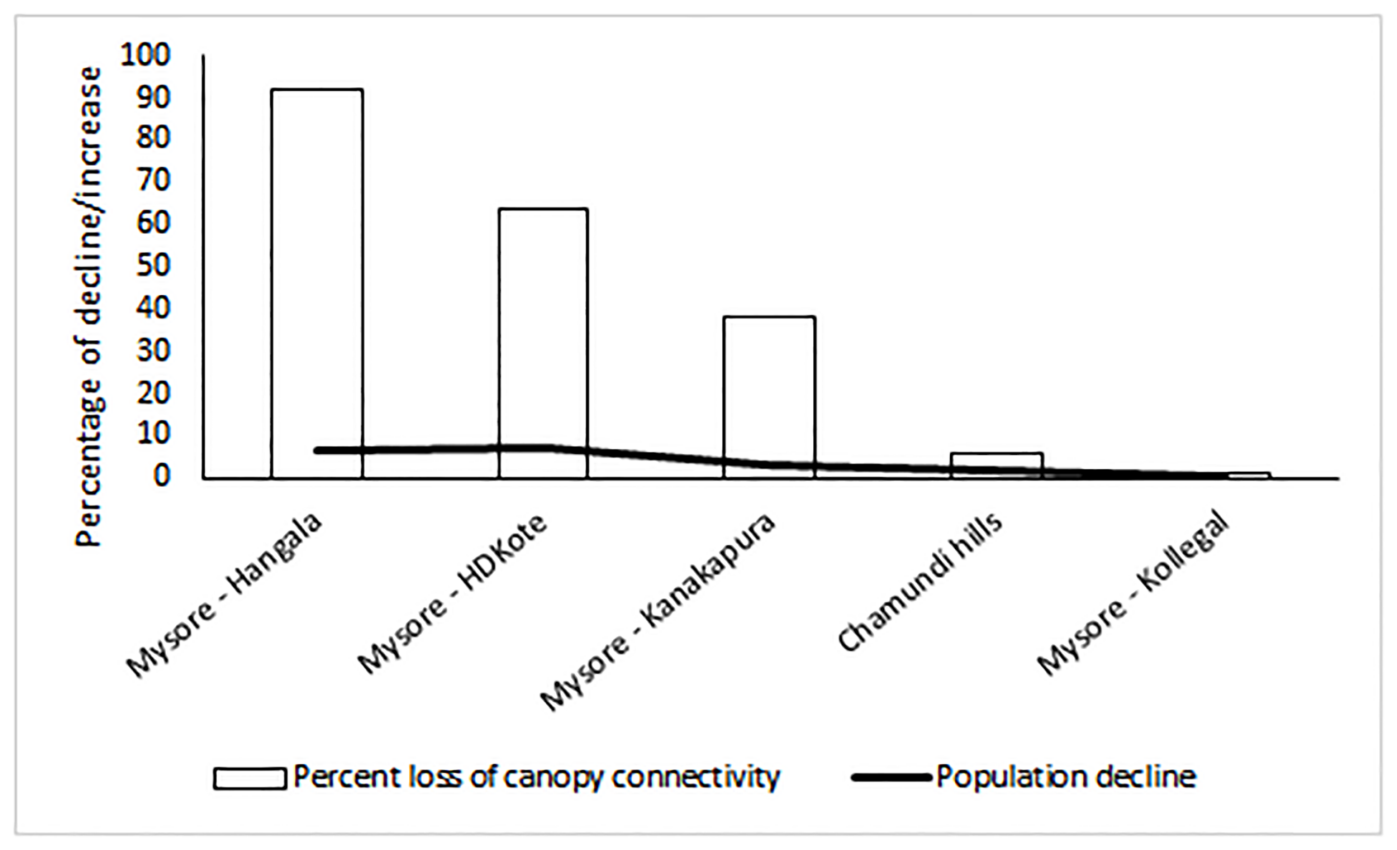
Canopy contiguity and percentage of annual population decline on the roadsides.

Percent canopy connectivity loss was observed more in Mysore-Ramnagar road (92%), followed by Mysore-Hangala road (92%), Mysore-HD Kote road (64%), Mysore-Kanakapura road (38%), Chamundi hill road (6%) and Mysore-Kollegal road (1%). Decline in canopy connectivity (%) was a good predictor of annual population decline (%) along the different roadsides between 2003 and 2015 (linear regression: R^2^ = 0.90, β = 0.07, P = 0.02; Fig 4).

#### 3.5.4 Land use change in areas around roadside habitats

**Fig 5:**
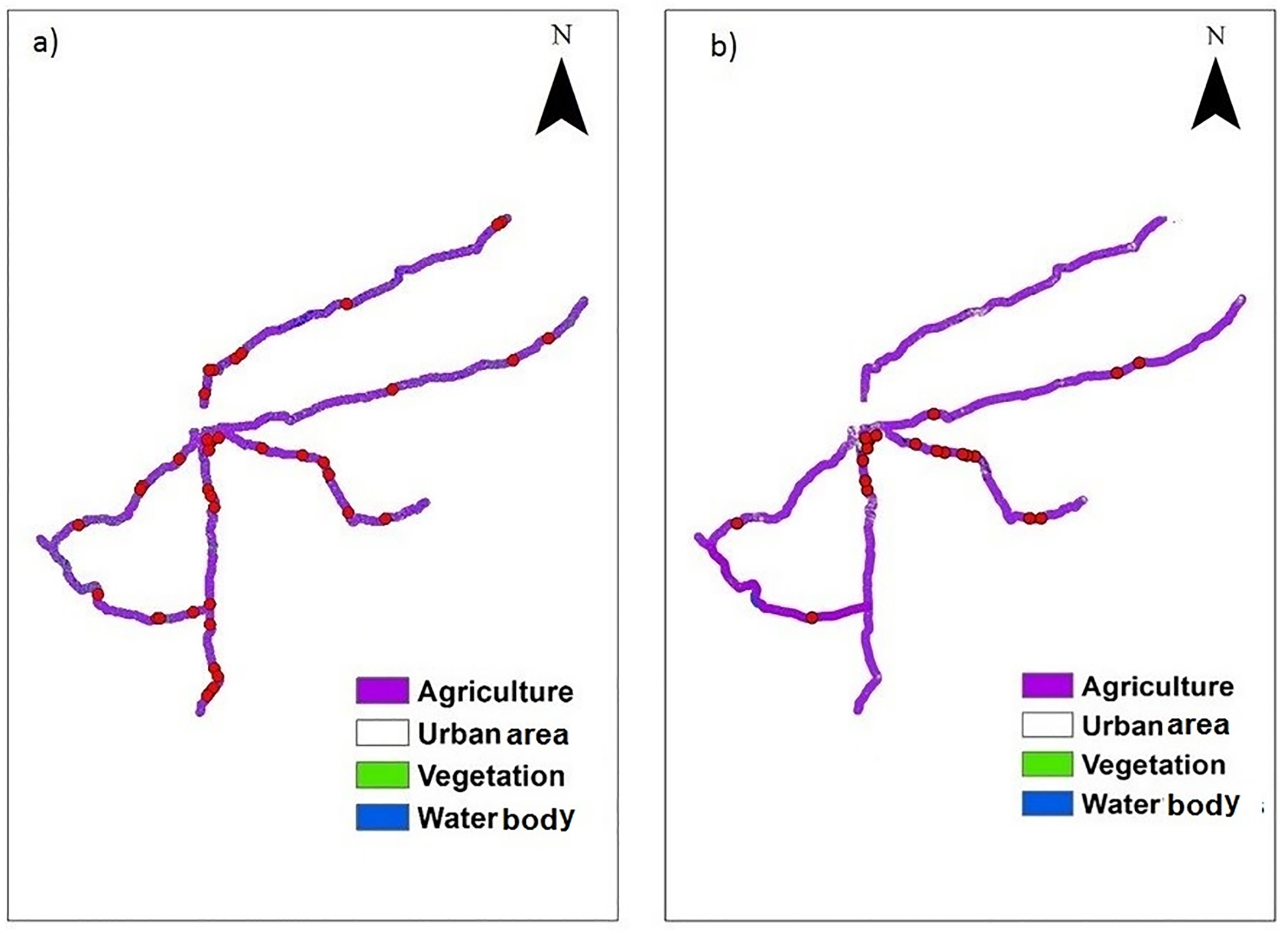
Land use in areas around different road side habitats and distribution of macaque groups along roadsides a) 2001 b) 2015.

Land use in areas around roadside habitats in 2001 was dominated by agricultural land (90%), followed by vegetation (6%), urban area (2%) and water bodies (2%). Land use in areas around the roadside habitats in 2015 was dominated by agricultural land (92%), followed by urban area (6%), vegetation (1%) and water bodies (1%). There was a decrease in the percent area covered by vegetation (Wilcoxons signed rank test: Z = -2.38, P = 0.02) and an increase in urban area (Wilcoxons signed rank test: Z = -2.37, P = 0.02) in areas around different roadside habitats between 2003 and 2015 (Table 8; Fig 5).

**Table 8:** Area covered (%) by each land use type around different roadside habitats.

Name of the road	Agricultural land (%)		Urban area (%)		Vegetation (%)		Water body (%)	
	2001	2015	2001	2015	2001	2015	2001	2015
Mysore-Ramnagar	93	93	1	6	5	0	1	1
Mysore-Kanakapura	92	96	1	3	6	0	1	1
Mysore-Kollegal	92	94	1	4	3	0	4	2
Mysore-Hangala	94	94	0	5	4	0	2	1
Mysore-HD Kote	91	93	1	6	7	0	1	1
Chamundi Hill	3	0	47	64	50	36	0	0
Begur-Handpost	90	95	0	1	7	0	3	4
Total	90	92	2	6	6	1	2	1

Due to these drastic habitat changes on and around the roads, the bonnet macaque populations have drastically declined and have no place to disperse as the vegetation in the surrounding agricultural fields has also reduced. Our fourth prediction that the number of bonnet macaques is drastically declining from the roadside habitats is also supported.

## 4. Discussion

We observed that the distributional range of the bonnet macaque is continually being reduced by the rhesus macaque in Peninsular India, the abundance of bonnet macaques in forested regions is very low, and the species is fast disappearing from its more common habitats around temples, tourist spots and roadsides.

Range extensions due to natural causes such as climate change have been reported in Protista [46], herbs, butterflies and birds [47] and small mammals [48]. Range extensions can also occur due to behavioural dispositions of the species. It has been shown that in sympatric congener rodents, a behaviourally more aggressive and hence dominant species could contribute to non-overlapping habitat associations [49]. Rhesus macaques have been classified into Grade 1, the most aggressive, of macaques whereas bonnet macaques are listed under Grade 3, a more tolerant species [50]. Grade 1 and Grade 2 macaque species are expected to have higher colonizing ability than the Grade 3 and Grade 4 species [50]. It has also been observed that the rhesus macaque with a larger body size and more aggressive temperament than the bonnet macaque displaced the latter from food and preferred habitats [20] if an encounter occurred between the two species. From the results of the previous [20] and the present study, it is observed that the rhesus macaque has invaded nearly 28,000 km^2^ range of the bonnet macaque in less than four decades. Whereas rhesus macaques occur in several countries from Afghanistan to Vietnam, the bonnet macaque is endemic to peninsular India. The displacement of bonnet macaques by rhesus macaques, therefore, could cause a serious conservation concern for the former in the long run.

Whereas some macaques such as the lion-tailed macaque are obligatory forest dwelling species, the bonnet macaque is largely commensal. Our data from wet forests as well as from other forest types show that the abundance of bonnet macaques in all forest types is very low, and detections are so few in several cases that even the standard models cannot be run. It indicates that the bonnet macaque is not a typically forest dwelling species. These monkeys have always occurred largely in temples, tourist places and on roadsides with surrounding agricultural lands. It has been observed that the animal species with a large brain size [51] and foraging innovations [52] can easily adapt to novel environments and new habitats which has been the case with the bonnet macaque of southern India. However, these habitats have been undergoing rapid changes over the past few decades. Recently there has been a tremendous increase in the number of people visiting temples and tourist places in India. It has been observed that the tolerance of people for macaques in such places has been decreasing and there have been frequent trappings and translocations of macaques from such places to unfamiliar habitats (MS—personal observations).

In the present study, we found that urbanization led to habitat (vegetation) loss and canopy connectivity loss along roadsides for the bonnet macaques and resulted in drastic population decline of the species. We also observed that bonnet macaques do not have any other habitats to migrate to, once roadside habitats are lost, since areas around roadsides are dominated by agricultural lands and urban areas which are unsuitable habitats.

Several studies [53–55]have shown that land use modifications by humans can lead to reduction in quality and quantity of food resources, and connectivity between canopies which in turn result in increase of mortality risk due to road accidents and predators, increased endoparasite load and bring a species in proximity with humans. A study on British urban mammals has shown how habitat fragmentation has negatively affected populations of these mammals [8]. Another study has shown how infrastructure has a negative impact on mammal abundance [14]. Proximity with humans may lead these animals to get into conflict with humans since they transmit diseases, damage buildings, food supplies and crops, cause traffic accidents and cause general nuisance by attacking, defecating and raiding for food [8]. Hence, we can infer that habitat loss and reduced canopy connectivity along roadsides might lead to several other associated risks for bonnet macaques. In such modified landscapes with several risk factors, local extinctions are most likely to occur [9]. We observed that one local roadside population of bonnet macaques became extinct and several others will be facing extinction in the near future. The bonnet macaques in coastal regions have almost been eliminated completely in those habitats [26].

Enquiries with farmers, villagers and devotees in temples, tourist spots and agricultural lands have revealed that they do not prefer to co-exist with bonnet macaques and they support translocation of the macaques [25]. Since our study covered a large area and large time span, our findings may also hold true for other macaque populations and other commensal species which are listed ‘least concern’, inhabiting roadsides and other human dominated habitats. Several other macaque species inhabit human dominated landscapes and are often in conflict with humans (e.g.: Rhesus macaque, *Macaca mulatta* [56]; Tonkean macaques, *Macaca tonkeana* [57]; Long-tailed macaques [58]). Under such circumstances, continuous monitoring of their population is necessary to assess their current status.

Conservation of small hillocks like Chamundi hills with good vegetation cover and some temples/tourist spots, which can presumably support viable populations of macaques, is a possible solution for conservation of populations of bonnet macaques as reserves. Our long-term data have also shown the population at Chamundi Hill to remain stable over 25 years whereas on the other roads, it has declined by 78%.Close to the city of Mysore, the Chamundi Hill Reserve Forest with an area of about 17 km^2^harbors many species of wild mammals including leopards *Panthera pardus*, wild pigs *Sus scrofa*, porcupines *Hystrix indica* etc. in addition to bonnet macaques (MS–Personal observations).We have described earlier [37] that India has innumerable such hillocks with reserved forests vegetation and Hindu temples and such areas can be actively managed for the conservation of commensal primate species. Establishment of new vegetation patches inside agricultural areas and creating corridors connecting them with roadsides, planting of fruiting trees on the sides of new roads, proper placement of new roads, building of artificial bridges across trees, creating awareness with farmers, villagers and devotees, and stopping unscientific unplanned translocations, will help in the conservation of this species, and several other species inhabiting roadside vegetation. As the bonnet macaque shares several traits such as commensalism, wide geographic distribution, seemingly large numbers etc. with many other commensal species that have also shown declining trends, our study can serve as a model for developing conservation strategies for all such species.

## Supporting information

S1 Table Group size of *M*. *radiata* and *M*. *mullata*. (DOCX)

S2 Table Detection of bonnet macaque and independent variables at Parambikulam. (XLSX)

S3 Table Predicted species response to each covariate for bonnet macaque. (DOCX)

S4 Table Detection probability for bonnet macaque. (DOCX)

S5 Table Model for occupancy for bonnet macaque at Parambikulam. (DOCX)

S6 Table Bonnet macaque sightings, their demography and land use characteristics on each road sectors of Mysore. (XLSX)
