# Supplementary material for: Losing its ground: A case study of fast declining populations of a ‘least-concern’ species, the bonnet macaque (Macaca radiata)
Source: PLoS One. 2017 Aug 23;12(8):e0182140. doi: 10.1371/journal.pone.0182140 (PMC5568106; doi:10.1371/journal.pone.0182140)
Supplement: S1 Table — (DOCX) [file pone.0182140.s001.docx]

**S1 Table. Group size of *M. radiata* and *M. mullata***

| No. | *M. radiata* | *M. mullata* |
| --- | --- | --- |
| 1 | 5 | 5 |
| 2 | 9 | 8 |
| 3 | 8 | 15 |
| 4 | 13 | 11 |
| 5 | 21 | 10 |
| 6 | 18 |  |
