# Supplementary material for: Losing its ground: A case study of fast declining populations of a ‘least-concern’ species, the bonnet macaque (Macaca radiata)
Source: PLoS One. 2017 Aug 23;12(8):e0182140. doi: 10.1371/journal.pone.0182140 (PMC5568106; doi:10.1371/journal.pone.0182140)
Supplement: S3 Table — (DOCX) [file pone.0182140.s003.docx]

**S3 Table: Predicted species response to each covariate for bonnet macaque**

| Covariates | $\psi$ | P |
| --- | --- | --- |
| KM | + | + |
| DUR | 0 | + |
| EG | + | 0 |
| ELE | - | 0 |
| CANO | + | 0 |
| DISTU | - | 0 |

KM: trail length; DUR: duration of the walk; EG: proportion of evergreen forests; ELE: elevation range; CANO: height of the canopy; DISTU: disturbance index. ‘+’ signifies a positive effect on the response variable, ‘_’ signifies a negative effect on the response variable and ‘0’ signifies that the covariate has no effect on the response variable. *ψ:* probability of occurrence; p, species detection probability
