# Supplementary material for: Losing its ground: A case study of fast declining populations of a ‘least-concern’ species, the bonnet macaque (Macaca radiata)
Source: PLoS One. 2017 Aug 23;12(8):e0182140. doi: 10.1371/journal.pone.0182140 (PMC5568106; doi:10.1371/journal.pone.0182140)
Supplement: S4 Table — (DOCX) [file pone.0182140.s004.docx]

**S4 Table: Detection probability for bonnet macaque**

| Model | $\hat{p}$ | AIC_c_ | ∆AIC_c_ | *w_i_* | *K* |
| --- | --- | --- | --- | --- | --- |
| $\psi$ (.), *p*(.) | 0.25 | 245.29 | 0.00 | 0.61 | 2 |
| $\psi$ (.), *p*(KM) | 0.28 | 247.37 | 2.08 | 0.22 | 3 |
| $\psi$ (.), *p*(DUR) | 0.28 | 249.11 | 3.82 | 0.09 | 3 |
| $\psi$ (.), *p*(KM+DUR) | 0.27 | 249.24 | 3.95 | 0.08 | 4 |

$\hat{p}$: is the estimated species detection probability; AICc: AIC corrected for small-sample bias; ∆AIC_c_: difference in AICc values between each model and the model with the lowest AICc; wi: AICc model weight; K: number of parameters estimated by the model. KM: trail length; DUR: duration of the walk
