# Supplementary material for: Losing its ground: A case study of fast declining populations of a ‘least-concern’ species, the bonnet macaque (Macaca radiata)
Source: PLoS One. 2017 Aug 23;12(8):e0182140. doi: 10.1371/journal.pone.0182140 (PMC5568106; doi:10.1371/journal.pone.0182140)
Supplement: S5 Table — (DOCX) [file pone.0182140.s005.docx]

**S5 Table: Model for occupancy for bonnet macaque at Parambikulam**

| Model | $\hat{\psi}$ | (S$\hat{E}$) | AIC_c_ | ∆AIC_c_ | *w_i_* | *K* |
| --- | --- | --- | --- | --- | --- | --- |
| $\psi$ (.), *p*(.) | 0.77 | 0.08 | 245.28 | 0.00 | 0.3973 | 2 |
| $\psi$ (ELE), *p*(.) | 0.50 | 0.06 | 248.05 | 0.00 | 0.1000 | 3 |
| $\psi$ (EG), *p*(.) | 0.49 | 0.06 | 248.32 | 0.27 | 0.0873 | 3 |
| $\psi$ (ELE+EG), *p*(.) | 0.49 | 0.09 | 248.90 | 0.85 | 0.0654 | 4 |
| $\psi$ (ELE+CANO), *p*(.) | 0.50 | 0.08 | 249.16 | 1.11 | 0.0574 | 4 |
| $\psi$ (CANO), *p*(.) | 0.50 | 0.05 | 249.33 | 1.28 | 0.0527 | 3 |
| $\psi$ (DISTU), *p*(.) | 0.50 | 0.05 | 249.81 | 1.76 | 0.0415 | 3 |
| $\psi$ (EG+ CANO), *p*(.) | 0.49 | 0.08 | 249.88 | 1.83 | 0.0400 | 4 |
| $\psi$ (DISTU+ELE), *p*(.) | 0.50 | 0.08 | 250.00 | 1.95 | 0.0377 | 4 |
| $\psi$ (ELE+ CANO +EG), *p*(.) | 0.49 | 0.11 | 250.15 | 2.10 | 0.0350 | 5 |
| $\psi$ (DISTU+EG), *p*(.) | 0.49 | 0.08 | 250.32 | 2.27 | 0.0321 | 4 |
| $\psi$ (DISTU+ELE+ CANO), *p*(.) | 0.50 | 0.11 | 251.15 | 3.10 | 0.0212 | 5 |
| $\psi$ (DISTU+ CANO), *p*(.) | 0.50 | 0.08 | 251.32 | 3.27 | 0.0195 | 4 |
| $\psi$ (DISTU+ELE+ CANO+EG), *p*(.) | 0.49 | 0.13 | 252.15 | 4.10 | 0.0129 | 6 |

$\hat{\psi:}$Estimated occupancy parameter; S$\hat{E}$: Associated standard error; AICc: AIC corrected for small-sample bias; ∆AIC_c_: difference in AICc values between each model and the model with the lowest AICc; wi: AICc model weight; K: number of parameters estimated by the model. KM: trail length; DUR: duration of the walk; EG: proportion of evergreen forests; ELE: elevation range; CANO: height of the canopy; DISTU: disturbance index
